# Animal Models for Human-Pathogenic Coronavirus and Animal Coronavirus Research

**DOI:** 10.3390/v17010100

**Published:** 2025-01-14

**Authors:** Fenglian Xiao, Jincheng Hu, Minsheng Xu, Di Wang, Xiaoyan Shen, Hua Zhang, Jie Miao, Haodong Cai, Jihui Wang, Yaqing Liu, Shan Xiao, Longchao Zhu

**Affiliations:** 1 School of Life and Health Technology, Dongguan University of Technology, Dongguan 523808, China; xiaofl@nfu.edu.cn (F.X.); hujc23@outlook.com (J.H.); xms111417@163.com (M.X.); diwang_cpu@163.com (D.W.); xyshen@dgut.edu.cn (X.S.); zhanghua@dgut.edu.cn (H.Z.); 18195161681@163.com (J.M.); z810347892@outlook.com (H.C.); wangjihui@dgut.edu.cn (J.W.); liuyaqing@dgut.edu.cn (Y.L.); 2 Traditional Chinese Medicine and Health School, Nanfang College, Guangzhou 510970, China; 3 Institute of Infectious Diseases, Shenzhen Bay Laboratory, Shenzhen 518132, China

**Keywords:** coronavirus, animal model, human, animal, infection, clinical manifestations

## Abstract

Coronavirus epidemics have posed a serious threat to both human and animal health. To combat emerging infectious diseases caused by coronaviruses, various animal infection models have been developed and applied in research, including non-human primate models, ferret models, hamster models, mouse models, and others. Moreover, new approaches have been utilized to develop animal models that are more susceptible to infection. These approaches include using viral delivery methods to induce the expression of viral receptors in mouse tissues and employing gene-editing techniques to create genetically modified mice. This has led to the successful establishment of infection models for multiple coronaviruses, significantly advancing related research. In contrast, livestock and pets that can be infected by animal coronaviruses provide valuable insights when used as infection models, enabling the collection of accurate clinical data through the analysis of post-infection pathological features. However, despite the potential insights, there is a paucity of research data pertaining to these infection models. In this review, we provide a detailed overview of recent progress in the development of animal models for coronaviruses that cause diseases in both humans and animals and suggest ways in which animal models can be adapted to further enhance their value in research.

## 1. Introduction

Coronaviruses, classified within the Orthocoronavirinae subfamily of the Coronavidae family, order Nidovirales, are enveloped, single-stranded, positive-sense RNA viruses. Since the emergence of severe acute respiratory syndrome coronavirus (SARS-CoV) in 2002, there has been heightened interest in coronaviruses. To date, seven human-pathogenic coronaviruses have been identified. The human coronaviruses (HCoVs) OC43, HCoV-HKU1, HCoV-229E, and HCoV-NL63 are commonly circulating strains that persist in human populations, typically causing mild-to-moderate respiratory infections [1,2,3,4]. The other three, SARS-CoV, Middle East respiratory syndrome coronavirus (MERS-CoV), and the newly emergent SARS-CoV-2, have the capacity to cause severe respiratory illnesses, posing a significant threat to public health. Other coronaviruses primarily circulate among animal populations, where they cause diseases ranging from mild to severe, and some may have zoonotic potential, making them capable of crossing species’ barriers.

**Figure 1:**
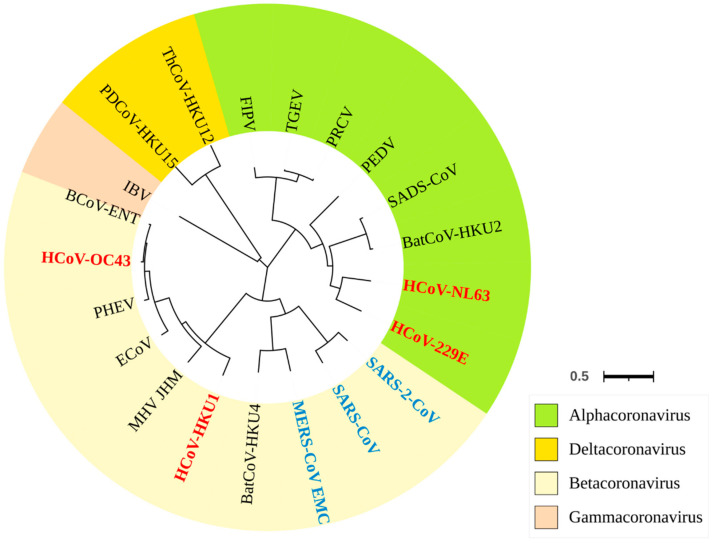
Phylogenetic tree of human and animal coronaviruses within the Coronavirinae subfamily. The tree was constructed using complete genomic nucleotide sequences and was generated via the Maximum Likelihood method under the GTR + I + G model using MEGA software version 11. The high and low human-pathogenic coronaviruses were highlighted by blue and red color, respectively. The tree was visualized with Interactive Tree of Life (iTOL) [8].

According to the International Committee on Taxonomy of Viruses (ICTV) (https://ictv.global, accessed on 30 October 2024), coronaviruses are currently categorized into four distinct genera: Alphacoronavirus, Betacoronavirus, Gammacoronavirus, and Deltacoronavirus. The coronaviruses that infect humans primarily belong to the Alphacoronavirus and Betacoronavirus genera. Specifically, HCoV-229E and HCoV-NL63 are classified under the Duvinacovirus and Setracovirus subgenera of Alphacoronavirus, respectively. In contrast, HCoV-OC43, HCoV-HKU1, SARS-CoV, MERS-CoV, and SARS-CoV-2 are classified under the Embecovirus, Merbecovirus, and Sarbecovirus subgenera of Betacoronavirus. Besides infecting humans, many members of the Alphacoronavirus and Betacoronavirus genera also infect various mammals (Figure 1). For instance, porcine coronaviruses such as porcine epidemic diarrhea virus (PEDV), transmissible gastroenteritis virus (TGEV), porcine hemagglutinating encephalomyelitis virus (PHEV), and porcine respiratory coronavirus (PRCV), along with the more recently identified swine acute diarrhea syndrome coronavirus (SADS-CoV), primarily infect pigs. Feline coronaviruses (FCoV), including feline infectious peritonitis virus (FIPV) and feline enteric coronaviruses (FECVs), infect cats, while murine coronaviruses such as murine hepatitis virus (MHV) infect mice. Gammacoronaviruses and Deltacoronaviruses are widespread globally and primarily infect wild and domestic birds. A well-studied example of a Gammacoronavirus is infectious bronchitis virus (IBV), which belongs to the Igacovirus subgenus. Additionally, porcine deltacoronavirus (PDCoV) HKU15, from the Buldecovirus subgenus within the Deltacoronavirus genus, has recently been identified in pigs [5,6,7].

Animal models are indispensable for understanding viral virulence, pathogenicity, and tissue tropism. They also play a crucial role in the development of drugs and therapeutics, providing vital insights into efficacy, safety, administration routes, pharmacokinetics, and pharmacodynamics. In the case of human-pathogenic coronaviruses, including SARS-CoV, MERS-CoV, and SARS-CoV-2, various animal models have been established and employed, proving instrumental in antiviral drug screening and vaccine development. Similarly, several models have been developed for coronaviruses responsible for animal diseases. In this review, we explore the pathogenesis of these coronaviruses and provide an overview of the animal models utilized in studies of coronavirus infections in both humans and animals.

## 2. Nonhuman Primate (NHP) Models

NHPs share a high degree of physiological, cognitive, neuroanatomical, social, reproductive, and developmental similarity with humans. With genetic homology to humans ranging from 75% to 98.5%, NHPs can often be infected by human coronaviruses. Numerous NHP models have been developed in recent years, particularly for studying highly pathogenic coronaviruses such as SARS-CoV, MERS-CoV, and SARS-CoV-2.

### 2.1. Rhesus Macaque

Studies have shown that SARS-CoV replicates within the respiratory tract of rhesus macaques, although the neutralizing antibody titters in serum are generally low, and the animals remain largely asymptomatic [9]. In contrast, rhesus macaques infected with SARS-CoV-2 exhibit a stronger inflammatory cytokine response and more severe lung lesions, indicating that this species is more susceptible to SARS-CoV-2 infection [10]. SARS-CoV-2 induces respiratory illness in rhesus macaques lasting 8 to 16 days, with high viral loads detected in nasal and throat swabs, as well as bronchoalveolar lavage fluid [10]. Infected rhesus macaques typically develop interstitial pneumonia, characterized by alveolar wall thickening and infiltration of monocytes and lymphocytes [11,12,13]. Additionally, lymphopenia, associated with severe infection in Coronavirus Disease 2019 (COVID-19) patients, has also been observed in these macaques [11,12]. Older rhesus macaques experience more severe SARS-CoV-2 infection compared to younger counterparts, with reduced antibody responses; increased viral shedding; slower viral clearance; and more pronounced lung lesions, weight loss, and pro-inflammatory cytokine production [14].

MERS-CoV infection in rhesus macaques typically results in transient lower respiratory tract infection accompanied by pneumonia. Elevated body temperature and reduced water intake are common within the first one to two days post-infection. Mild clinical symptoms are usually observed early in the course of infection, with viral shedding, replication in respiratory tissues, and gene expression peaking in the initial stages before gradually declining. MERS-CoV induces multifocal, mild-to-moderate interstitial pneumonia, with viral replication primarily localized in alveolar cells. Despite the presence of the virus in the lungs, no viral dissemination to other organs, such as the kidneys, trachea, brain, heart, liver, spleen, or intestines, has been observed [15,16].

### 2.2. Common Marmoset

When infected with SARS-CoV, common marmosets develop mild clinical symptoms, such as diarrhea and dyspnea. Most infected animals exhibit monocytic interstitial pneumonia, characterized by multinucleated syncytial cells, edema, and bronchiolitis. Viral antigens are primarily localized in alveolar macrophages and type-I pneumocytes. Hepatic inflammation, manifested as multifocal lymphocytic hepatitis with single-cell necrosis, is common, along with mild diffuse colitis and multifocal lymphocytic myocarditis (Table 1) [17,18]. In contrast, common marmosets show lower susceptibility to SARS-CoV-2 infection, with minimal or undetectable levels of viral RNA in swab samples and tissues (Table 2) [13,19,20].

In MERS-CoV-infected marmosets, disease characteristics similar to those observed in human cases have been documented, including renal and liver dysfunction [53]. Clinical symptoms include rapid and shallow breathing, dyspnea, cyanosis, and hemorrhagic oral secretions. Severe respiratory distress is common, and high viral loads are found in the lungs, accompanied by widespread bronchointerstitial pneumonia. Viable virus is isolated from pharyngeal swabs and lung tissue. In addition, virus antigen can be detected in T cells of infected marmosets [54]. In animals challenged with the MERS-CoV strain England 1 via the intranasal route, small acute inflammatory foci are observed in the lungs, along with expanded alveolar septa, neutrophil infiltration, and mild exudation into the alveolar spaces [55]. A combination of ocular, oral, tracheal, and intranasal inoculation of MERS-CoV results in severe, often fatal, respiratory disease in marmosets (Table 3).

### 2.3. African Green Monkeys

African green monkeys (AGMs) infected with SARS-CoV exhibited mild symptoms. Moderate-to-high SARS-CoV titters accompanied by interstitial pneumonitis were observed in the lungs of AGMs at 2 dpi, with resolution by 4 dpi. The virus replicated in the lung, tracheal, or turbinate tissues, with pulmonary infection presenting as focal interstitial mononuclear inflammation and pulmonary edema. Immunohistochemistry revealed focal viral antigen distribution in tracheal and bronchiolar epithelial cells, lung cells, and macrophages at early stages [8]. Similarly, SARS-CoV-2 infection in AGMs leads to mild respiratory illness, with replication predominantly confined to the lower respiratory tract, where macrophages contribute to a pro-inflammatory state in the lungs (Table 1) [64]. At 3 dpi, AGMs inoculated with active SARS-CoV-2 showed varying degrees of pulmonary lesions and mediastinal lymph node enlargement. At 10 dpi, alveolar edema, type II pneumocyte hyperplasia, increased alveolar macrophages, and infiltration of lymphocytes and neutrophils were observed, along with proliferative nodules associated with terminal airways (Table 2) [34,65]. In MERS-CoV models, AGMs infected with aerosolized virus exhibited mild clinical respiratory signs, including chest congestion, rales, or wheezing, but did not develop severe disease. Across low-to-high doses of MERS-CoV infection, no significant weight loss was observed throughout the study. But significantly higher viral titters were detected in throat swab specimens and serum samples. All AGMs exposed to aerosolized MERS-CoV developed multifocal interstitial pneumonia with mild or minor lung lesions by 28 dpi (Table 3) [56].

### 2.4. Cynomolgus Macaque

Cynomolgus macaques (Macaca fascicularis) have been widely used as models for studying SARS-CoV and SARS-CoV-2 infections. Infected macaques show mild-to-moderate symptoms, including reduced appetite, decreased activity, mild coughing, sneezing, and slight respiratory distress, mirroring the milder syndromes observed in children with SARS-CoV infections. Viral replication is detected in multiple tissues, showing a tropism for upper respiratory mucosa, although viremia is not a prominent feature, and viral RNA levels in nasal and oral swabs are generally low [57,66]. Older cynomolgus macaques are more susceptible to SARS-CoV infection than younger adults, showing higher viral RNA loads and more severe lung lesions, paralleling age-related differences observed in human cases of SARS (Table 1) [21,22].

Although SARS-CoV infection in cynomolgus macaques does not fully recapitulate the severe disease observed in human adults, this model has proven useful for studying mechanisms of severe COVID-19. In SARS-CoV-2-infected cynomolgus macaques, a greater proportion of lung tissue is affected, with interstitial pneumonia, endothelialitis, elevated inflammatory cytokine levels, and reduced lymphocytic infiltration observed [11]. Viral replication is also detected in respiratory tissues, as well as in the ileum, colon, mesentery, and tonsils. Infected elderly macaques demonstrate higher viral loads in nasal swabs, reflecting the greater susceptibility of older humans to SARS-CoV-2. However, no significant clinical symptoms or weight loss are typically observed in SARS-CoV-2-infected macaques (Table 2) [19,35].

## 3. Mouse Models

### 3.1. Wild-Type Mouse Models

It is generally accepted that ancestral MERS-CoV and SARS-CoV-2 do not infect wild-type mice [67,68,69]. However, wild-type mice become susceptible to SARS-CoV-2 when the virus acquired the N501Y mutation in the spike protein. This mutation is found in Alpha, Beta, Gamma, and all Omicron subvariants [36,70,71,72]. In mouse-adapted SARS-CoV-2 (MASCp6 strain), both aged and young BALB/c mice developed mild-to-moderate pneumonia [70]. Infection with B.1.1.7 and other N501Y-carrying variants resulted in high viral titters and genome copies detectable in the nasal turbinates and lungs of wild-type mice, with viral shedding in the respiratory tract for approximately 4 to 7 days [71].

Human coronaviruses HCoV-229E and HCoV-NL63 are unable to establish infections in wild-type mice, whereas HCoV-OC43 infection in wild-type mice depends on the inoculation route, viral dose, age, and strain. When administered intracerebrally, HCoV-OC43 shows low virulence in neonatal mice, with clinical symptoms such as tremors, stiffness, and lethargy; however, virulence increases with repeated passages [73]. Once HCoV-OC43 reaches the central nervous system (CNS), infected mice develop acute encephalitis due to neuronal infection. Viral RNA is detectable in the brain as early as 24 h post-infection (hpi) and in the spinal cord by 2 to 3 dpi [74,75]. Some neurons undergo apoptosis during the acute phase, and surviving mice may develop motor impairments linked to persistent viral presence in the CNS and neuronal loss in the brain [74,76].

### 3.2. Aged Mouse Models

Previous studies have demonstrated that susceptibility to SARS-CoV infection in wild-type mice is age-dependent. For instance, earlier reports suggest that although 4-to-6-week-old BALB/c mice support viral replication in their respiratory tracts following intranasal SARS-CoV infection, they remain asymptomatic, with no lung lesions, and the virus is cleared within a week [23]. In contrast, intranasal SARS-CoV infection in 12-to-14-month-old BALB/c mice results in significant weight loss, hunching, ruffled fur, mild dehydration, and lung inflammation. Additionally, the virus was detected in the upper respiratory tract and even the liver of aged BALB/c mice, reflecting the heightened susceptibility to SARS-CoV infection observed in elderly humans [24]. A mouse-adapted SARS-CoV strain (MA15 virus) was developed through serial lung passaging in BALB/c mice, and this strain proved lethal to young (6–8-week-old) BALB/c mice. The viral titters in the lungs of MA15-infected mice were 1000 times higher than those in SARS-CoV-infected mice at 24 hpi and remained elevated through 4 dpi, surpassing the peak levels seen in SARS-CoV-infected mice [77].

SARS-CoV-2 replicates more efficiently in the respiratory tracts of aged mice than in young mice following viral exposure. SARS-CoV-2 variants with the N501Y mutation in the spike protein can infect wild-type C57BL/6N mice, and intranasal inoculation with the B.1.1.7 variant led to no visible signs of disease in young mice, while aged mice displayed ruffled fur, a hunched posture, and labored breathing, with the symptoms peaking at 4 dpi. SARS-CoV-2 infection caused more severe inflammatory damage to lung tissue in aged mice than in young mice [78]. SARS-CoV-2 MA infection in aged mice resulted in epithelial damage, peribronchial lymphocyte inflammation, increased hemorrhage, and edema at 2 dpi and 4 dpi, with viral antigens detected in the conducting airway epithelium, interstitium, and nasal epithelium [79]. Additionally, aged mice infected with SARS-CoV-2 MA10 exhibited increased morbidity and mortality [80]. The SARS-CoV-2 B.1.351 variant infection had a lethal impact on aged BALB/c mice. Infection of aged BALB/c mice (older than 6 months) with the SARS-CoV-2 B.1.351 variant (strain TY8-612) induced a prolonged and elevated production of pro-inflammatory cytokines/chemokines, leading to multi-organ damage and ultimately resulting in widespread mortality [81].

### 3.3. Disease Mouse Models

Intranasal infection of male SCID mice with 10^5^ TCID_50_ of the Beta (B.1.351) variant led to high viral loads in the lungs and moderate lung pathology by 3 dpi. The infectious viral titters in the lungs of SARS-CoV-2 Beta variant-infected SCID mice were significantly higher at 3 dpi compared to concurrently infected immunocompetent BALB/c (1 log10 higher) or C57BL/6 mice (1.2 log10 higher), with most mice showing persistent viral loads in the lungs up to 7 dpi [82]. Obesity has been identified as an independent risk factor for severe outcomes in COVID-19 and other infectious diseases. Studies show that diet-induced obese mice infected with SARS-CoV-2 variants experience more severe tissue damage and significant weight loss compared to lean mice, and neutralizing antibodies are undetectable in diet-induced obese mice [83,84,85]. Diabetic mice infected with SARS-CoV-2 exhibit prolonged lung damage and elevated serum inflammation. Moreover, diabetic mice infected with SARS-CoV-2 show greater insulin resistance and increased loss of pancreatic islet cells compared to uninfected diabetic mice [86]. Diabetic C57BL/6J mice with leptin receptor gene deficiency show higher viral loads in the throat and lungs and slower viral clearance in the throat post-infection compared to C57BL/6J mice. Pre-existing cardiovascular disease and diabetes mellitus could exacerbate the histopathological changes in pneumonia during SARS-CoV-2 infection [87].

### 3.4. Transgenic Mouse Models

Transgenic mouse models are useful for understanding the molecular pathogenesis mechanisms of viruses that are previously not susceptible to wild-type mice. They are also frequently used for comparative studies of pathogenic differences in SARS-CoV-2 and its variants [88,89,90]. To develop susceptible mouse models for viral infections, various strategies, such as generating transgenic mice and receptor-transduced mice, have been widely employed. In vitro analysis of the binding affinity between viral spike (S) proteins and receptors revealed that the binding affinity of SARS-CoV and SARS-CoV-2 S proteins to mouse angiotensin-converting enzyme 2 (mACE2) is 63.1% and 0.8%, respectively, compared to human ACE2 (hACE2) [91]. To address this affinity limitation, the transgenic K18-hACE2 mouse model was created, where the hACE2 gene is driven by the human cytokeratin 18 (K18) promoter, allowing expression of hACE2 in the epithelial cells of mice. K18-hACE2 mice infected with SARS-CoV developed severe clinical symptoms, such as significant weight loss, lethargy, labored breathing, and death by 7 dpi [25,92]. Some K18-hACE2 mice infected with SARS-CoV-2 also developed CNS infections, with viral loads and pathology in the brain being undetectable in the early stages but rapidly increasing by 4–5 dpi, ultimately leading to death [37,92,93].

Other transgenic mouse models have been generated using different promoters to express hACE2. For instance, Menachery et al. cloned hACE2 under the ciliated airway epithelial cell-specific HFH4/FOXJ1 promoter, creating HFH4/FOXJ-hACE2 transgenic mice on a C3H × C57BL/6 background that are susceptible to both SARS-CoV and SARS-CoV-2 [26,38]. After SARS-CoV-2 infection, these mice exhibit interstitial pneumonia and pathologies similar to those seen in COVID-19 patients, including significant weight loss, lymphopenia, and elevated creatine kinase levels [38]. Another model, the CAG-hACE2 transgenic mice, which use a synthetic CAG promoter for ubiquitous hACE2 expression, displayed acute lung injury early in infection due to a marked increase in cytokine and chemokine levels; however, the mic exhibited atypical histopathological features (Table 4) [27,39,94,95].

However, species differences in ACE2 distribution outside the lungs may also affect systemic responses to SARS-CoV-2 infection. For example, mACE2 expression is highly localized to the bronchial epithelium, whereas hACE2 is more broadly distributed in human lungs [97,98]. To better replicate human ACE2 expression patterns in mice, Zhang et al. recently developed a new genome-editing approach to generate humanized ACE2 mice. These mice express hACE2 regulatory elements and splice isoforms at levels similar to human expression, offering a more physiologically relevant model for COVID-19 research. The ACE2 GREAT-GEMM mice exhibit human-specific transcription and splicing patterns and are susceptible to SARS-CoV-2 infection after intranasal challenge, but they do not succumb to infection, making them a more accurate model for studying human COVID-19 compared to K18-hACE2 mice [40].

In addition, researchers have used CRISPR/Cas9 technology to produce susceptible mice by editing viral entry receptor genes. Sun et al. employed CRISPR/Cas9 knock-in technology to replace mACE2 with hACE2 in wild-type C57BL/6 mice, generating a stable hACE2-expressing mouse model to study SARS-CoV-2 infection. High viral loads were detected in the trachea, lungs, and brains of both young and aged hACE2 knock-in mice, with older mice developing interstitial pneumonia [96].

In the MERS-CoV transgenic mouse model, through CRISPR/Cas9 to replace two amino acids at positions 288 and 330 in mouse dipeptidyl peptidase 4 (mDPP4) with their human counterparts enabled efficient MERS-CoV infection and replication in the lungs, with infected mice developing severe acute respiratory distress syndrome (ARDS)-like symptoms [58,59]. After intranasal inoculation with MERS-CoV in hDPP4 transgenic mice, the mice exhibited systemic inflammation, ranging from mild-to-severe pneumonia, along with liver, kidney, and spleen damage, characterized by neutrophil and macrophage infiltration. Clinically, hDPP4 transgenic mice infected with MERS-CoV showed reduced activity and significant weight loss starting from day 6 post-infection, with all infected mice succumbing by day 10. MERS-CoV infection in the central nervous system (CNS) triggers complement activation, resulting in inflammation-mediated damage to brain tissue, manifesting as high viral loads and paralysis of virus-positive neurons [99,100]. Human exons 10–12 were substituted for mouse DPP4 exons 10–12 to create human DPP4 receptor knock-in mice. Through continuous passage, the resulting adapted viruses induced lethal pulmonary diseases, including diffuse alveolar damage and immune dysregulation [101].

### 3.5. Adenovirus 5 Receptor Expression Mouse Models

To render mice susceptible to the respective coronaviruses, rapid expression of hACE2 or human DPP4 (hDPP4) has been employed, resulting in infected mice displaying symptoms and pathology similar to those observed in human patients. C57BL/6 and BALB/c mice transduced with adenovirus encoding hDPP4 became susceptible to MERS-CoV infection, developing early signs of perivascular and peribronchial lymphocytic infiltration in the lungs, followed by interstitial pneumonia [60]. Using a similar method, Sun et al. recently generated hACE2-transduced mice, which exhibited efficient viral replication in the lungs and severe lung lesions following SARS-CoV-2 infection. These mice showed clinical symptoms, including ruffled fur, hunching, labored breathing by 2 dpi, and around 20% body weight loss by 4–6 dpi [41]. In humanized hACE2 NOD-SCID IL2Rγ^−/−^ (NIKO) mice infected with SARS-CoV-2, pathological damage, such as inflammatory cell infiltration and tissue injury, was observed in the lungs, making this model useful for studying the pathology of SARS-CoV-2 within a human-like immune environment and for evaluating potential therapies (Table 5) [42].

Mouse models for HCoV-229E and HCoV-NL63 have also been developed through adenoviral delivery of human aminopeptidase N (hAPN) and hACE2. Expression of hAPN, the receptor for HCoV-229E, is necessary but not sufficient for susceptibility in vivo. HCoV-229E infection in mice has been facilitated by crossing hAPN transgenic mice with Signal Transducer and Activator of Transcription (Stat) 1-deficient mice and adapting the HCoV-229E virus to grow in hAPN transgenic, Stat1-deficient fibroblasts [103]. These mice only develop mild diseases, with all hAPN^+/+^Stat1^−/−^ mice surviving at least 18 dpi. Clinically, susceptible mice exhibited slight weight loss and a mild rise in body temperature, and organs from hAPN^+/+^Stat1^−/−^ mice challenged through various routes showed hemorrhagic areas in the lungs and small intestines, particularly between 2 and 4 dpi [102]. Mice deficient in the type I interferon receptor (IFNAR^−/−^) or STAT1^−/−^ that were sensitized with Ad5-hAPN or Ad5-hACE2 developed pneumonia characterized by inflammatory cell infiltration, with viral clearance observed by 7 dpi. IFNAR^−/−^ C57BL/6 mice transduced with Ad5-hAPN and infected with HCoV-229E lost 5–10% of their body weight in the first 4 dpi, with high viral loads detected in lung tissue that gradually decreased over the course of infection. In contrast, Ad5-hAPN-transduced IFNAR^−/−^ BALB/c mice lost up to 15% of their body weight, with higher viral titers in the lungs, suggesting that the BALB/c genetic background confers greater susceptibility to 229E infection [103].

## 4. Other Animal Models

### 4.1. Ferret

Ferrets are not susceptible to MERS-CoV and do not support its replication [104]. However, they are highly susceptible to SARS-CoV and SARS-CoV-2 infections [28,43,105]. Upon SARS-CoV infection, the virus is detectable in the lungs, and infected ferrets show lung tissue damage, although clinical symptoms are generally mild, including lethargy without significant weight loss [28]. Viral replication in the lungs, trachea, and nasal turbinates peaks on day 5 or 6, reaching levels of 10^6^ TCID50/mL in lung homogenates [29]. Histopathologically, ferrets display multifocal lung lesions affecting 5–10% of the lungs, with mild alveolar damage and lymphocytic infiltration around the bronchioles and blood vessels [28,105].

Ferrets infected with SARS-CoV-2 exhibit symptoms similar to human COVID-19, including elevated body temperature, reduced activity, decreased appetite, and coughing, lasting from 2 to 12 dpi [43,44]. Histological analysis shows severe perivascular and lymphoplasmacytic vasculitis in the lungs by 13 dpi [43]. Ferrets of different ages respond differently to SARS-CoV-2 infection; for example, 1-to-2-year-old ferrets experience prolonged fevers, while younger ferrets show minimal fever. Three-year-old ferrets exhibited fevers lasting beyond 10 dpi, with greater weight loss and slower recovery compared to younger animals. Older ferrets shed higher viral loads in respiratory secretions and feces compared to their younger counterparts [45].

Ferrets are particularly useful in viral transmission studies due to their cough reflex, which mice and rats lack [106,107]. SARS-CoV-2 is efficiently transmitted between ferrets via direct contact and airborne routes (through respiratory droplets and/or aerosols) [46]. In transmission studies, some juvenile ferrets exposed to infected ferrets through direct or indirect contact tested positive without showing symptoms, indicating effective airborne transmission [43].

### 4.2. Hamster

Hamsters display a relatively high degree of homology with hACE2, differing by only two contact residues, making them highly susceptible to infection by SARS-CoV and SARS-CoV-2 [108]. However, despite high levels of DPP4 expression, hamsters, like mice, are not susceptible to MERS-CoV infection [109]. Upon infection with SARS-CoV (Urbani strain at 10^3^ or 10^5^ TCID_50_), hamsters develop productive infections peaking on 2–3 dpi in the nasal turbinates and lungs, with viral clearance by 7 dpi. The infection also causes extrapulmonary dissemination, including transient viremia and spread to organs such as the liver and spleen. Pulmonary histopathology shows interstitial inflammation and focal consolidation by 3 dpi, progressing to more extensive consolidation affecting 30–40% of the lungs by 7 dpi. Despite these significant lung lesions, hamsters do not exhibit marked clinical illness or mortality [30,31,32].

In contrast, hamsters infected with SARS-CoV-2 experience significant weight loss and show viral replication in the nasal mucosa and lower respiratory tract epithelial cells. Infection of olfactory sensory neurons in the nasal mucosa likely explains the anosmia (loss of smell) commonly reported in COVID-19 patients. Infected hamsters exhibit viral replication in the lungs, with histological evidence of lung edema, inflammation, and lesions associated with cell death, contributing to weight loss. Hamsters also efficiently support the transmission of SARS-CoV-2 via direct contact or aerosol routes [47,48,49,110].

The hamster model is instrumental for studying the virology and pathogenesis of SARS-CoV-2, particularly in relation to emerging variants. For example, the D614G substitution in the spike protein significantly increases viral transmissibility, while the N501Y substitution enhances viral replication in the upper respiratory tract and improves transmission. The P681R substitution has been shown to increase pathogenicity, and the R203K/G204R dual substitution in the nucleocapsid protein improves viral adaptability compared to parental strain. In one study by Dhakal et al., male hamsters exhibited higher infection rates and more severe lung damage than female hamsters, with males showing greater weight loss [111,112]. Notably, the SARS-CoV-2 Omicron variant demonstrated lower lung infectivity and pathogenicity in hamsters compared to the Delta and B.1.1 variants, consistent with observations in human patients [113,114,115].

### 4.3. Mink

Minks are highly susceptible to SARS-CoV-2 infection, developing significant weight loss, respiratory distress characterized by infiltrative pneumonia, and, in some cases, death. Infected minks exhibit mucopurulent secretions in their nasal cavities, with viral RNA detected in multiple respiratory and extra-respiratory tissues, including the turbinates, soft palate, tonsils, all lung lobes, submandibular lymph nodes, and trachea [50]. SARS-CoV-2 is also widely distributed in other organ systems, including cardiovascular, hepatobiliary, urinary, endocrine, digestive, and immune systems, leading to multi-organ and systemic lesions. These pathological changes are associated with significantly increased inflammatory responses, resembling those observed in critically ill COVID-19 patients. Moreover, SARS-CoV-2-infected minks display lipidomic and metabolomic alterations similar to severe COVID-19 cases in humans, suggesting potential therapeutic applications of melatonin against COVID-19.

### 4.4. Rat

The cotton rat is widely used as a model for studying respiratory viral infections, including human parainfluenza, human rhinovirus, and respiratory syncytial virus [116,117,118]. However, intranasal inoculation with MERS-CoV or SARS-CoV did not result in detectable viral loads in the tissues of infected rats, indicating that rats may not be suitable for studying MERS-CoV and SARS-CoV infections [119,120]. In contrast, SARS-CoV-2 infection in cotton rats has been shown to have a multisystemic impact, affecting multiple organs and physiological systems. The highest viral loads were detected in the turbinates of S. fulviventer cotton rats, while lower levels were found in the lungs and salivary swabs, with minimal differences observed between species [121]. In Sprague-Dawley (SD) rats, intranasal inoculation with SARS-CoV-2 leads to successful infection, resulting in viral replication in both the upper respiratory tract and lungs. Despite this, none of the infected rats exhibited overt clinical symptoms. Gross necropsy revealed mild lung lesions, including edema and sporadic punctate hemorrhages [122]. Although rats may not be ideal models for respiratory infections like SARS-CoV and MERS-CoV, they are well-suited for studying demyelinating encephalomyelitis caused by murine coronaviruses [123]. For instance, intracerebral inoculation of Lewis rats with the murine coronavirus MHV-JHM typically results in acute encephalitis, with the majority of animals succumbing to infection within 14 days [124]. Additionally, infection with MHV demonstrates age-dependent pathogenicity in Wistar Furth rats. Intracerebral inoculation of 2-day-old rats leads to acute encephalitis, while infection in 10-day-old rats results in a chronic demyelinating disease characterized by progressive posterior paralysis [125,126].

### 4.5. Dog and Cat

While dogs are sensitive to SARS-CoV-2, they typically experience only mild infections. Infected dogs do not shed the virus but do seroconvert and develop neutralizing antibodies [51]. Cats, however, can act as intermediate hosts or viral carriers, increasing the risk of zoonotic transmission between humans and animals. Viral antigens have been detected in the epithelial cells of the turbinates, necrotic debris in the tonsils, submucosal glands of the trachea, and intestinal enterocytes. Transmission studies show that viral RNA can be found in the soft palate and tonsils of inoculated cats, as well as in the turbinates, soft palate, tonsils, and trachea of exposed cats. Histopathological analysis of young cats, which died or were euthanized by 3 days post-inoculation, revealed extensive lesions in the nasal and tracheal mucosa, as well as in the lungs. These findings indicate that SARS-CoV-2 replicates efficiently in cats, with younger cats being more susceptible than older ones [43].

Feline Infectious Peritonitis (FIP) is a severe and typically fatal disease caused by variants of FCoV. While FCoV is relatively common among feline species, most cats infected with this virus do not develop FIP because they are able to mount an effective immune response to combat the virus. However, in certain cases, the virus may mutate, or the host’s immune system may fail to effectively control the infection, leading to the development of FIP. FIPV predominantly affects cats under three years of age in multi-cat environments, such as shelters and breeding facilities. Neurological forms of FIPV often present with non-specific symptoms, including weight loss, fever, and lethargy. Abdominal abnormalities, such as mesenteric lymph node lesions and irregularities in the spleen and kidneys, are common in cats with neurological FIPV [127,128,129]. Additionally, affected cats may display neurological symptoms such as dementia, pica, seizures, and incontinence [130].

### 4.6. Pig

Studies have shown that pigs exhibit low sensitivity to infection with SARS-CoV, SARS-CoV-2, and MERS-CoV [43,131]. However, pigs are hosts for several other coronaviruses that can cause severe diarrhea and death, particularly in piglets (Table 6). One of the major viruses is TGEV, which causes diarrhea and high mortality in seronegative newborn piglets. TGEV primarily replicates in the epithelial cells of the small intestine’s villi, leading to villous atrophy and malabsorptive diarrhea [132,133]. In contrast, PRCV, a variant of TGEV, replicates in the respiratory tract rather than the intestines, typically resulting in subclinical infections or mild respiratory illness [134,135,136,137,138,139]. PEDV also poses a significant threat to pig populations, with recent years seeing the emergence of more virulent PEDV strains. PEDV infects the entire gastrointestinal tract, including the jejunum, ileum, duodenum, and the cecum/colon, with high virulence particularly in the small intestine [140,141]. Microminipigs used in PEDV infection studies have exhibited severe villous atrophy in the small intestines; however, the large intestines remain unaffected [142]. Recently, bat-derived coronaviruses like SADS-CoV and PDCoV-HKU15 have emerged in pig farms. SADS-CoV, particularly infective in piglets, causes watery diarrhea, rapid weight loss, and severe villous atrophy [142,143]. In contrast, PDCoV-HKU15 results in milder clinical symptoms, such as persistent diarrhea [144,145,146,147].

### 4.7. Chicken

Chickens are not susceptible to SARS-CoV-2 infection, but they are a useful model for studying IBV, a common avian coronavirus. IBV causes respiratory symptoms, including sneezing, wheezing, coughing, and nasal discharge. The severity of the disease varies by age, with younger chickens being more severely affected than adults [149,150].

### 4.8. Bat

Bats are natural reservoirs for many zoonotic viruses, including coronaviruses. Studies involving the infection of North American big brown bats and Egyptian fruit bats with SARS-CoV-2 have shown species-specific responses. North American big brown bats are resistant to SARS-CoV-2 infection, while Egyptian fruit bats show transient infection without significant clinical symptoms or histopathological changes [151,152,153].

### 4.9. Camelid

Members of the Camelidae family, such as camels, llamas, and alpacas, are susceptible to MERS-CoV. Dromedary camels, in particular, show significant infection in the nasal turbinates and trachea, with viral RNA detectable in these tissues and immune cell infiltration at 4 dpi [61,62].

### 4.10. Raccoon Dog

Raccoon dogs are susceptible to SARS-CoV-2 infection and can transmit the virus to other animals under farm-like conditions. Infected raccoon dogs shed viral RNA from nasal and rectal swabs for up to 16 days but show no clinical illness [52].

### 4.11. Masked Palm Civet

Masked palm civets, known as potential intermediate hosts for SARS-CoV, are susceptible to infection, with the virus detectable in multiple organs, including the lungs, liver, kidneys, and heart. SARS-CoV can be found in throat and anal swabs from 3 to 18 dpi, and viral RNA has been detected in lymph nodes and spleen even at the end of experimental periods [33].

### 4.12. Guinea Pig

Guinea pigs have been tested as models for SARS-CoV infection, but their suitability is limited. Infected guinea pigs show a mild temperature increase, and no significant virus replication or lung pathology has been observed [154].

### 4.13. Rabbit

New Zealand white rabbits can serve as an asymptomatic model for MERS-CoV infection. Although they show no clinical signs, mild heterophilic infiltration is observed in the nasal cavity and lungs, with hypertrophy of type II pneumocytes [63].

### 4.14. Horse

Horses can be used as a model for equine coronavirus (ECoV) infection, which causes fever and anorexia. ECoV infections in horses are generally short-lived but result in consistent viral shedding in feces [155].

## 5. Discussion

When addressing emerging infectious diseases like SARS-CoV, MERS-CoV, and SARS-2-CoV, establishing effective animal models is crucial for understanding viral pathogenic mechanisms. These models not only accelerate vaccine and antiviral drug development but also help scientists better comprehend viral transmission patterns. Given the highly mutable nature of coronaviruses, animal models have been essential for assessing the pathogenesis of coronavirus variants, particularly SARS-CoV-2, and for uncovering subtle differences in pathogenicity across different models [156,157,158,159]. Animal models play an indispensable role in this process, providing a platform for researchers to study the interactions between viral mutations and host responses. However, not all animal models can precisely replicate human infection conditions. Each model has its limitations and reflects only specific pathogenic characteristics. Thus, careful selection of the appropriate model is essential to ensure the validity and reliability of experimental results, depending on the research objectives.

There are relatively few animal models available for studying low-pathogenic coronaviruses. Although low-pathogenic coronaviruses generally cause mild symptoms in humans similar to the common cold, most individuals experience only minor illness. However, immunocompromised individuals may still develop severe, potentially life-threatening respiratory diseases.

Coronaviruses that cause diseases in economically important animals and pets display clear pathogenic characteristics in their hosts, providing researchers with established disease models. These models simplify the study of viral pathogenic mechanisms and facilitate the development of vaccines and antiviral therapies. By leveraging these naturally occurring disease models, scientists can accelerate the translation of laboratory research into practical applications, thus safeguarding animal health and minimizing economic losses.

Although large animals possess all the necessary traits for infection models, small animal models, such as mice, remain indispensable in research due to cost efficiency and ease of use. Mice offer rich genetic diversity and are easily genetically modified, making them ideal for in-depth studies of viral pathogenic mechanisms. Therefore, despite the advantages of larger models, small animal models continue to be crucial tools in research. Future studies will require the ongoing development of suitable animal models to gain deeper insights into coronavirus pathogenic mechanisms, transmission routes, and potential health threats.

## Figures and Tables

**Table 1:** Animal models used in SARS-CoV research.

Models	Virus Replication	Clinical Signs	Severity	Applications	References
Rhesus macaque	Upper and lower respiratory tract	Asymptomatic infection	Mild	Antivirus drug screening; vaccine development	[9]
African green monkey	Lung, tracheal, nasal turbinate, and intestinal	Fever; hemoglobin concentrations and hematocrit decrease	Mild	Antivirus drug screening; vaccine development	[9]
Cynomolgus macaque	Mainly on lower respiratory tract, low levels of replication in spleen, and cervical and bronchial lymph nodes	No fever, coughing, lethargy, diarrhea, and dyspnea	Mild to moderate; more severe in the older	Antivirus drug screening; vaccine and pathogenesis study	[9,21,22]
Common marmoset	Lung and liver	Increase in rectal temperature, and diarrhea	Mild	Antivirus drug, vaccine, and pathogenesis study	[17]
Young mouse	N/A	No clinical signs in young mice	Mild	Pathology	[23]
Aged mouse	Lung, nose, liver, and spleen	Weight loss, hunching, ruffled fur, slight dehydration, and lung inflammation	Moderate	Antivirus drug, vaccine, and pathogenesis study	[23,24]
Transgenic mouse	Lung, brain, and nose	Weight loss, lethargy, labored breathing, and death	Severe	Antivirus drug, vaccine, and pathogenesis study	[25,26,27]
Ferret	Respiratory tract, gastrointestinal tract, and urinary tract	Lethargy, conjunctivitis, sneezing, fever, diarrhea, mortality, and morbidity	Severe	Virus transmission, antivirus drug screening, and pathogenesis	[28,29]
Hamster	Lung, nasal turbinate, liver, and spleen	No significant clinical signs	Severe	Virus transmission, antivirus drug screening, vaccine development, and pathogenesis	[30,31,32]
Palm civets	Throat, rectum, lungs, liver, kidneys, and heart	Fever	N/A	Cross-species transmission	[33]

**Table 2:** Animal models used in SARS-CoV-2 research.

Models	Virus Replication	Clinical Signs	Severity	Applications	Reference
Rhesus macaque	Nose and lung	Weight loss, reduced appetite, dehydration, coughing, and irregular respiration patterns	More severe in the older	Antivirus drug screening, vaccine development, and pathogenesis	[10,11,12,13,14]
African green monkey	Nearly all tissues	Decreased appetite, fever, transient lymphocytopenia, and thrombocytopenia	Severe	Antivirus drug screening, vaccine development, and pathogenesis	[34]
Cynomolgus macaque	Nose, throat, rectal, and lungs	Little or no symptoms	Moderate	Antivirus drug screening, vaccine development, and pathogenesis	[11,14,19,35]
Common marmoset	Lung, nose, and respiratory tract	Little or no fever symptoms	Mild	Pathogenesis	[14,20]
Wild-type mouse	Lung, spleen, and kidney	Weight loss	Moderate in aged mouse and young mice infected by the mouse-adapted strain	Antivirus drug screening, vaccine development, and pathogenesis	[36]
Transgenic mouse	Lung, trachea, turbinalia, liver, brain, spleen, kidney, small intestine, and heart	Weight loss, respiratory distress, neurological symptom, and death	Severe	Antivirus drug screening, vaccine development, and pathogenesis	[37,38,39,40]
Viral Receptor Expression Mouse by Adenoviral Delivery	Lung	Weight loss and tachypnea	Mild	Antivirus drug screening and pathogenesis	[41,42]
Ferret	Lung, nose, and rectum	Fever, anorexia, ruffled fur, snoring, and coughing	Moderate	Virus transmission, antivirus drug screening, and pathogenesis	[28,43,44,45,46]
Hamster	Lung, nose, intestine, and kidney	Weight loss, lethargy, tachypnea, ruffled fur, and hunched posture	Severe	Virus transmission, antivirus drug screening, vaccine development, and pathogenesis	[47,48,49]
Mink	Nasal turbinate, soft palate, tonsil, lung, submandibular lymph nodes, trachea, urinary tract, and kidney	Weight loss	Severe	Virus transmission	[50]
Dog	Rectum	No significant symptoms	Mild	Virus transmission	[43,51]
Cat	Nasal turbinate, trachea, intestine, and soft palate	N/A	N/A	Virus transmission	[43]
Raccoon Dog	Nose, throat, and rectum	Mild rhinitis	Mild	Pathogenesis	[52]

**Table 3:** Animal models used in MERS-CoV research.

Models	Virus Replication	Clinical Signs	Severity	Applications	Reference
Rhesus macaque	Lung, nasal, oropharyngeal, urogenital, bronchus, and rectal	Lower respiratory tract infection, pneumonia, elevated temperatures, and reduced water intake	Mild	Antivirus drug screening and vaccine development	[15,16]
African green monkey	Throat, liver, and lung	Higher respiratory rates in high virus dose, chest congestion, and fever	Mild	Antivirus drug screening and vaccine development	[56]
Cynomolgus macaque	Nose, throat, rectal, lungs, and spleen	No clinical signs	Mild	Antivirus drug screening and vaccine development	[19,57]
Common marmoset	Lung, kidney, nasal, and throat	Fever, dyspnea, loss of appetite, and lethargy	Moderate to severe	Antivirus drug screening and vaccine development	[53,55]
Transgenic mouse	Lung	Weight loss, severe respiratory distress	Severe	Antivirus drug screening; vaccine and pathogenesis study	[58,59]
Viral Receptor Expression Mouse by Adenoviral Delivery	Lung	Weight loss	Moderate	Drug screening and pathogenesis study	[60]
Dromedary Camels	Upper respiratory tract	A slight fever	Mild	Transmission	[61,62]
Rabbits	Lung and nose	No clinical signs	Mild	Transmission	[63]

**Table 4:** Transgenic mouse models for human-pathogenic coronavirus.

Viruses	Models	Virus Replication	Clinical Signs	Severity	Applications	Reference
SARS-CoV	K18-hACE2	Lung and brain	Weight loss, lethargy, labored breathing, and death	Severe	Antivirus drug screening; vaccine development	[25]
	HFH4/FOXJ1-hACE2	Lung and brain	Rapid weight loss and death	Severe	Antivirus drug screening; vaccine development	[26]
	CAG-hACE2	Lung, brain, and nose	Weight loss, acute wasting syndrome, ruffled fur, lethargy, tachypnea, and death	Severe	Antivirus drug screening; vaccine development	[27,94]
SARS-CoV-2	K18-hACE2	Lung, brain, kidney, spleen, gastrointestinal tract, and nasal turbinates	Marked weight loss, rhinitis, death	Severe	Antivirus drug screening; vaccine development	[37,92,93]
	HFH4/FOXJ1-hACE2	Lung, brain, eye, and heart	Weight loss, marked respiratory distress, neurological symptom, and death	Severe	Antivirus drug screening; vaccine development	[38]
	CAG-hACE2	Lung, trachea, turbinalia, liver, brain, spleen, kidney, small intestine, and heart	Dramatic weight loss, and death	Moderate to severe	Antivirus drug screening; vaccine development	[39,95]
	ACE2 GREAT-GEMM mice	Lung	N/A	Mild	Antivirus drug screening; vaccine development	[40]
	hACE2 knock-in mouse	Lung, trachea, and brain	N/A	Mild	Antivirus drug screening; vaccine development	[96]
MERS-CoV	hDPP4 transgenic mouse	Lung	Weight loss and severe respiratory distress	Severe	Antivirus drug screening; vaccine development	[58,59]

**Table 5:** Viral receptor expression mouse by adenoviral delivery for human-pathogenic coronavirus.

Viruses	Models	Virus Replication	Clinical Signs	Severity	Applications	Reference
SARS-CoV-2	Ad5-hACE2-transduced mouse	Lung	Weight loss and tachypnea	Mild	Antiviral drug evaluation; vaccine development	[41]
	hACE2 NOD-SCID IL2Rγ^−/−^ (NIKO) mice	Lung	No clinical signs	Mild	Disease model; pathogenesis study	[42]
MERS-CoV	Ad5-hDPP4-transduced mouse	Lung	Weight loss	Moderate	Antiviral drug evaluation; vaccine development	[60]
HCoV-229E	hAPN^+/+^Stat1^−/−^ mouse	Lung, spleen, intestine, liver	Slight weight loss and fever	Mild	Antiviral drug evaluation; vaccine and antibody development	[102]
	Ad5-hAPN transduced IFNAR^−/−^ mouse	Lung	Weight loss	Mild	Antiviral drug evaluation; vaccine and antibody development	[103]
HCoV-NL63	Ad5-hACE2 transduced IFNAR^−/−^ mouse	Lung	Weight loss	Mild	Antiviral drug evaluation; vaccine and antibody development	[103]

**Table 6:** Other models used for animal coronaviruses research.

Viruses	Models	Virus Replication	Clinical Signs	Severity	Applications	Reference
TGEV	Pig	Lung, spleen, and intestine	Diarrhea, anorexia, vomiting, and high mortality in piglets	Severe in piglets	Vaccine and antivirus drug evaluation; pathogenesis	[132,133]
PRCV		Respiratory tract and gut	No clinical signs	Mild	Pathogenesis	[137,138,139]
PEDV		Small intestine and gastrointestinal tract	Weight loss, watery diarrhea, vomiting, high mortality in piglets	Severe in piglets	Vaccine and antivirus drug evaluation; pathogenesis	[140,141,148]
SADS-CoV		Intestine	Weight loss, diarrhea, and high mortality in piglets	Severe in piglets	Vaccine and antivirus drug evaluation; pathogenesis	[142]
PDCoV-HKU15		Respiratory tract and intestine	Dehydration, weight loss, lethargy, and death	Severe in piglets	Vaccine and antivirus drug evaluation; pathogenesis	[144,145,146,147]
IBV	Chicken	Respiratory tract and kidney	Sneezing, wheezing, coughing, conjunctivitis, difficulty breathing, and diarrhea	Moderate to severe	Vaccine and antivirus drug evaluation; pathogenesis	[149,150]
FIPV	Cat	Eyes, brain, and kidney	Lethargy, seizures, incontinence, compulsive licking, fever, and weight loss	Mild to severe	Vaccine and antivirus drug evaluation; pathogenesis	[51,127,128]
